# Online NCHHSTP Atlas Updated with County-Level Data

**Published:** 2013-09-20

CDC’s National Center for HIV/AIDS, Viral Hepatitis, STD, and TB Prevention (NCHHSTP) recently updated its NCHHSTP Atlas by adding county-level data for human immunodeficiency virus (HIV) and sexually transmitted infections in the United States. The atlas, an interactive, online mapping tool and platform for accessing data collected by NCHHSTP, allows users to observe disease trends and patterns of HIV, acquired immunodeficiency syndrome (AIDS), certain sexually transmitted infections (i.e., chlamydia, gonorrhea, primary and secondary syphilis, and early latent syphilis), tuberculosis, and acute viral hepatitis A, B, and C. The atlas also allows users to create detailed reports, maps, and other graphics based on these surveillance data. The NCHHSTP Atlas is available at http://www.cdc.gov/nchhstp/atlas.

